# The Lack of Nutritional Counseling during Hospitalization

**DOI:** 10.5935/abc.20190172

**Published:** 2019-08

**Authors:** Luiza Antoniazzi

**Affiliations:** Instituto do Coração (InCor) - Lipids, São Paulo, SP - Brazil

**Keywords:** Hospitalization, Healthy Diet, Risk Reduction Behavior, Inpatients, Dietetics

During the nutritional follow-up of hospitalized individuals, the nutritionist has as activities to perform the diagnosis, dietary prescription, supervise the distribution of the diets and evaluate their acceptance, and also perform nutritional counseling so that these individuals understand how a standard specific diet may be more appropriate taking into account their diagnoses and nutritional status. The number of nutritionists issued by Resolution 600 of 2018 of the Federal Council of Nutritionists is 1 professional every 15 beds of high complexity and every 30 beds of medium complexity.^1^

Lima et al.^2^ evaluated in an article published in this edition whether nutritional counseling was performed in the hospital environment for patients with Acute Myocardial Infarction (AMI) and the quality of this orientation. The authors found that 57.6% of the individuals hospitalized in the public network and 70.3% private hospitals, both in Sergipe, Brazil, had received in-hospital nutritional counseling.

One possible cause of this low rate of counseling provided may be the amount of nutritionists available in hospital institutions, which is lower than predicted by the resolution.^1^

Seta et al.,^3^ 2010, evaluated 8 public hospitals in 4 Brazilian states, of which none of the nutritionists evaluated reported nutritional counseling.^3^

Another problem is the quality of the guidance provided. It should be checked whether it meets the guidelines for preventing the occurrence of new cardiovascular events.

In the cited article there was a predominance of restrictive guidelines, especially salt and fat. About the insertion of cardioprotective foods, patients from the private network were more benefited, mainly regarding the consumption of fruits and vegetables.

The diet for prevention after acute myocardial infarction requires caloric adequacy, applied calorie restriction when necessary for the adequacy of nutritional status. It is important that the macronutrients are adequate within normality, taking into account the restriction of saturated fats and balance between the other fats as recommended by the dyslipidemia guideline.^4-6^ In Addition, current guidelines on prevention of cardiovascular events recommend a diet similar to the Mediterranean diet, salt intake of < 5 g per day; 30-45 g fibre per day; regular consumption of fruits and vegetables per daily; regular consumption of fish and unsalted nuts daily; limited alcohol intake; and discouraging sugar-sweetened drinks.^7,8^

The better understanding of the food behavior is indispensable to deepen the knowledge of the determinants of the alimentary behavior, which include a complex range of nutritional, demographic, social, cultural, environmental and psychological factors. Several studies point out that the transthoracic model,^9^ developed by two US researchers, James O. Prochaska and Carlo DiClemente,^10^ in the 1980s, can be considered a promising instrument to help understanding health-related behavioral change, and is widely used in research and clinical practice. The transthoracic model of behavior change presents 5 stages. In the pre-contemplation stage, it has not yet been considered by the individual or no changes were made to the behavior and there is no intention to adopt them in the near future. In the stage of contemplation, the individual begins to consider behavioral change. That is, it is intended to change the behavior in the future, but a deadline has not yet been set, therefore.

The decision-making individual, also called the preparation, intends to change his behavior in the near future, as in the next month. Generally, after overcoming previous attempts frustrated, small changes are made and a plan of action is adopted, still not making a serious commitment to the same. Already the individuals in action correspond to those who have in fact altered their behavior, their experiences or their environment so as to overcome barriers previously perceived. Such changes are visible and have occurred recently, as in the last six months. In the maintenance stage, the individual already changed his behavior and kept him more than six months.^10^

The study by Vieira et al.,^11^ carried out with individuals after angioplasty at a hospital specialized in cardiology, in São Paulo, identified the stages of behavior change in which they were. 36% were in maintenance, 26% in preparation, 17% in pre-contemplation, 12% in action and 9% in contemplation. It is necessary for the nutrition team to create adequate food education strategies for the individuals at each stage, in order to promote adherence to a more favorable food plan and the adequacy of nutritional status.

A study with individuals from the northern region of Paraná after AMI or angioplasty aimed at identifying changes in attitudes and habits in these survivors. The main changes identified were an increase in the number of meals, an increase in fruit consumption, a reduction in the consumption of fats and fried foods, and the use of the salt shaker on the table. The number of patients who did not perform physical activity decreased.^12^

It is very important to evaluate the achievemente and the quality of nutritional counseling to allows the implementation of appropriate actions, since the moment after the recent cardiovascular event may favor the adoption of favorable dietary changes for these individuals.
